# N-actylcysteine inhibits diethyl phthalate-induced inflammation via JNK and STAT pathway in RAW264.7 macrophages

**DOI:** 10.1186/s12860-025-00537-9

**Published:** 2025-04-16

**Authors:** Jin Hee Kim, Jae Hoon Lee, Yoon Jung Koo, Jong Wook Song

**Affiliations:** 1 Department of Anesthesiology and Pain Medicine, Yonsei University College of Medicine, 50 Yonsei- ro, Seodaemun-gu, Seoul, 03722 Republic of Korea; 2 Anesthesia and Pain Research Institute, Yonsei University College of Medicine, 50 Yonsei-ro, Seodaemun-gu, Seoul, 03722 Republic of Korea

**Keywords:** Diethyl phthalate, Nacetyl cysteine, Inflammation, Cyclooxygenase-2, c-Jun N-terminal kinases

## Abstract

**Background:**

Phthalates are plasticizers that cause inflammation in several cell types and adversely affect the health of humans and animals. Nacetylcysteine (NAC) has been shown to exert antioxidant effects in various diseases. However, the effect of NAC on diethyl phthalate (DEP)-induced toxicity in macrophages has not yet been elucidated. In this study, we investigated the effect and underlying mechanisms of NAC on DEP-induced inflammation in RAW264.7 macrophages. RAW264.7 macrophages were pretreated with NAC for 2 h followed by exposure to DEP. We investigated the effect of NAC on NO, reactive oxygen species (ROS), prostaglandin E2 (PGE2), and glutathione (GSH) levels following DEP exposure. In addition, pathway-related genes including cyclooxygenase-2 (COX-2), inducible nitric oxide synthase (iNOS), mitogen-activated protein kinase (MAPK), and signal transducer and activator of transcription (STAT) were evaluated using western blot.

**Results:**

Treatment with 100 and 300 µM DEHP, DBP, and DEP significantly increased the protein levels of cyclooxygenase-2 (COX-2) and inducible nitric oxide synthase (iNOS) compared with those in the control group. However, NAC pretreatment downregulated the levels of NO, PGE2, and ROS, elevated GSH levels, and suppressed the mRNA levels of inflammatory cytokines such as interleukin (IL)-1β, IL-6, COX-2, and iNOS compared with those in the DEP-treated group. In addition, NAC significantly reduced the levels of p-JNK and p-STAT1/3 in RAW264.7 macrophages treated with DEP.

**Conclusions:**

NAC pretreatment inhibits DEP-induced inflammation via the MAPK/JNK and STAT1/3 pathways in macrophages.

**Supplementary Information:**

The online version contains supplementary material available at 10.1186/s12860-025-00537-9.

## Background

Phthalate, a widespread environmental pollutant, is of serious concern owing to its potentially toxic effects, including reproductive toxicity [1], neurotoxicity [2], and carcinogenicity [3]. Phthalates constitute a class of endocrine disruptors, which are used to increase the flexibility of polyvinyl chloride (PVC) products [4], and are commonly used as plasticizers or solvents in various consumer products such as infant care products and toys, beauty products, medical devices, and the plastic coating of some oral medications [5]. Phthalate exposure occurs through ingestion, inhalation, or intravenous or dermal contact. Commonly used phthalates include diethyl phthalate (DEP), di-n-butyl phthalate (DBP), diisobutyl phthalate (DiBP), butyl benzyl phthalate (BBzP), di(2-ethylhexyl) phthalate (DEHP), and diisononyl phthalate (DiNP) [6].

Research has shown that over 80% of human urine samples contain detectable phthalates [7, 8]. Additionally, human metabolic studies indicate that the average intake of various phthalates, including DBP, DIBP, and DEHP, ranges from 0.3 to 3 µg/kg/day [9]. Moreover, women are prone to increased exposure to phthalates due to using beauty products containing phthalate chemicals [10]. Notably, phthalate exposure has been reported to be higher in children than in adults [11]. Further, DBP and DEHP exposure during pregnancy increases fetal resorption and incidence of external malformations in mouse offspring [12].

The mechanisms underlying phthalate toxicity involve oxidative stress and inflammation, which are key processes in the progression of various diseases [13]. Oxidative stress is caused by a disturbance of reactive oxidative species (ROS), which results in DNA damage, lipid peroxidation of membranes, oxidative modification of proteins, and apoptosis [14]. ROS generation depends on the NADPH oxidase holoenzyme, which generates superoxide ions. Inflammation refers to a complex physiological response to harmful stimuli, such as pathogens or irritants, caused by macrophage activation and is involved in the development of various diseases. Activated macrophages induce inflammation by producing oxidative stress mediators. Thus, macrophages play an important role in tissue repair through phagocytosis, innate immune function, and activation and resolution of inflammation [15].

Urinary phthalate metabolites are associated with indicators of urinary oxidative stress. Several cell types, including neutrophils, also produce ROS following phthalate exposure [16]. Considering that phthalate exposure induces oxidative stress, effective strategies must be identified to inhibit phthalate-induced oxidative stress, with RAW264.7 macrophages serving as a widely used cell type for studying the cellular effects of chemicals and particles. Recent findings suggest that Nacetylcysteine (NAC) demonstrates antioxidant effects in various diseases. Therefore, in this study, we aimed to investigate the effect and underlying molecular mechanisms of the effect of NAC on DEP-induced inflammation in RAW264.7 macrophages. Specifically, we evaluated the extent to which NAC pretreatment ameliorates DEP-induced inflammation in macrophages by decreasing COX-2/PGE2 via the MAPK/JNK and STAT pathways.

## Methods

### Materials

DBP, DEP, DEBP, celecoxib (CCXB), indomethacin (INDO), and NAC were purchased from SigmaAldrich (St. Louis, MO, USA). Phospho-ERK, phospho-JNK, phospho-p38, ERK, JNK, p38, phospho-STAT1, phospho-STAT3, STAT1 and STAT3 antibodies were obtained from Cell Signaling Technology (Danvers, MA, USA). Anti-COX-2 and anti-iNOS antibodies were purchased from Abcam (Cambridge, UK). Anti-β-actin antibody was obtained from Santa Cruz Biotechnology (Dallas, TX, USA).

### Cell culture

RAW264.7 macrophages were purchased from the American Type Culture Collection (ATCC, Manassas, VA, USA). Cells were cultured in Dulbecco’s modified Eagle’s medium (DMEM) supplemented with 10% fetal bovine serum (FBS; HyClone, Logan, UT, USA) at 37 °C in a humidified atmosphere (5% CO_2_ and 95% air). Prior to experiments, cells were plated in 96-well (1.5 × 10^4^ cells per well) or 6-well (5 × 10^6^ cells per well) plates for 24 h and then incubated with an inhibitor or 100 and 300 µM of phthalates for 24–48 h. In each experiment, treatments were performed in triplicate.

### Phthalates

The phthalate mixture was dissolved in dimethyl sulfoxide (DMSO) and diluted to achieve the desired phthalate concentrations of 100 and 300 µM. The concentrations used in this study were based on previous reports; measurements of phthalate monoester levels in human plasma indicate that environmental exposure can reach 0.4 µM and occupational and medical procedure exposures can reach 40 µM [17, 18]. Although above the human exposure range, previous in vitro studies used 400 µM as the highest concentration [19–21]. Therefore, DEP concentrations of 100 and 300 µM were used in the present study.

### Inhibitor treatment

To investigate the potential inhibitory effects of COX inhibitors and antioxidants on phthalate-induced oxidative stress, RAW264.7 cells were pretreated with or without CCXB (10 µM), INDO (10 µM), or NAC (10 mM) for 2 h before incubation with phthalates. Thereafter, proteins were harvested from the cells via trypsin digestion and centrifugation.

### NO measurement

Culture media were collected and centrifuged at 1,500 rpm and 4 °C for 10 min. The Griess Reagent System (Promega) determined NO levels in the supernatant. Briefly, 50 µL of sulfanilamide reagent and 50 µL of 0.1% naphthalene ethylenediamine reagent (NED) were added to 50 µL of supernatant from each well, followed by incubation in the dark for 15 min at room temperature to measure NO levels. The absorbance was measured at 550 nm using a microplate reader. Sodium nitrite was used to prepare a standard curve.

### Measurement of ROS production

To determine ROS levels, cells were seeded in plates and treated with NAC and DEP. After washing with serum-free medium, 10 µM of DCFDA-Cell ROS Assay Buffer (Abcam, Cambridge, UK) was added to each well, and the plate was incubated at 37 °C for 30 min. The fluorescence intensity was measured using a microplate reader at excitation and emission wavelengths of 485 and 535 nm, respectively. The images were obtained using a fluorescence microscope (200x magnification).

### GSH/GSSG determination

Cells were seeded onto clear 96-well microtiter plates at a density of 1 × 10^5^ cells/well. Cell lysates were analyzed for glutathione using a GSH/GSSG assay kit (Promega Corporation, Madison, WI, USA). The GSH/GSSG ratio was calculated according to the manufacturer’s instructions. Luminescence was measured with a microplate reader, and the GSH/GSSG ratio was calculated as [(net treated total glutathione RLU − net treated GSSG RLU)/(net treated GSSG RLU)] × 2, where RLU is relative light units.

### Reverse transcription–quantitative polymerase chain reaction

Total RNA was extracted from cells using an RNeasy Mini kit (Qiagen, Valencia, CA, USA) and reverse-transcribed to generate cDNA using CycleScript RT PreMix (Bioneer, Daejeon, South Korea). Quantitative real-time RT–PCR was performed using SYBR Green PCR Master Mix (Applied Biosystems, Foster City, CA, USA) and specific primers (Table 1). *COX-2*, *iNOS*, *IL-1β*, and *IL-6* mRNA levels were normalized to that of *GAPDH*.

**Table 1:** Primers used for qPCR

Gene	Sequence (5′ → 3′)
*COX-2*	Forward: CCACTTCAAGGGAGTCTGGA Reverse: AGTCATCTGCTACGGGAGGA
*iNOS*	Forward: TCAGCCACCTTGGTGAAGGGAC Reverse: TAGGCTACTCCGTGGAGTGAACA
*IL-1β*	Forward: TGACGGACCCCAAAAGATGA Reverse: TCTCCACAGCCACAATGAGT
*IL-6*	Forward: CTCTCCGCAAGAGACTTCCAG Reverse: TGTTGTGGGTGGTATCCTCTG
*TNF-α*	Forward: CGTCGTAGCAAACCACCAAG Reverse: GGCAGAGAGGAGGTTGACTT
*GAPDH*	Forward: CCCATCACCATCTTCCAGGAGC Reverse: CCAGTGAGCTTCCCGTTCAGC

### Western blot analysis

Cells were lysed in RIPA buffer (Cell Signaling Technology) containing a protease inhibitor cocktail (cOmplete™ Mini Protease Inhibitor Tablet; Roche Diagnostics GmbH, Mannheim, Germany) to extract the proteins. Protein concentrations were determined using a Pierce BCA Protein Assay kit (Thermo Fisher Scientific, Waltham, MA, USA). Proteins (30 µg) were separated on 8–16% and 6% SDS–PAGE gels (Bio-rad) and transferred to nitrocellulose membranes (Thermo Fisher Scientific). After blocking with 5% (w/v) skim milk (Bio-rad) for 1 h at room temperature, the membranes were incubated overnight at 4 °C with primary antibodies against COX-2 (1:1,000), iNOS (1:1,000), p-Akt (1:1,000), p-JNK (1:1,000), and β-actin (1:5,000). Thereafter, the membranes were washed with Tris-buffered saline containing 0.1% Tween 20 and incubated with secondary horseradish peroxidase-conjugated anti-rabbit/mouse IgG antibodies (1:5000; Thermo Fisher Scientific) for 2 h at room temperature. Protein bands were visualized using a Pierce-enhanced chemiluminescence substrate (Thermo Fisher Scientific) and quantified using ImageJ software. Protein expression was normalized to that of β-actin.

### Enzyme-linked immunosorbent assay

The levels of IL-1β and PGE2 in the culture supernatant were assessed using a commercially available enzyme-linked immunosorbent assay (ELISA) kit (Abcam and R&D Systems, Minneapolis, MN, USA), according to the manufacturer’s instructions.

### Statistical analysis

All statistical analyses were performed using SPSS Windows software (version 22.0; SPSS Inc., Chicago, IL, USA). Quantitative data are presented as the mean ± standard error of the mean from three experimental replicates. Significant differences between groups were determined using two-tailed Student’s *t*-test (two groups) or one-way analysis of variance with Tukey’s post hoc comparisons (multiple groups). Statistical significance was set at *p* < 0.05 (*p < 0.05 and **p < 0.01).

## Results

### DEHP, DBP, and DEP exposure on inflammatory cytokines in RAW264.7 macrophages

**Fig. 1:**
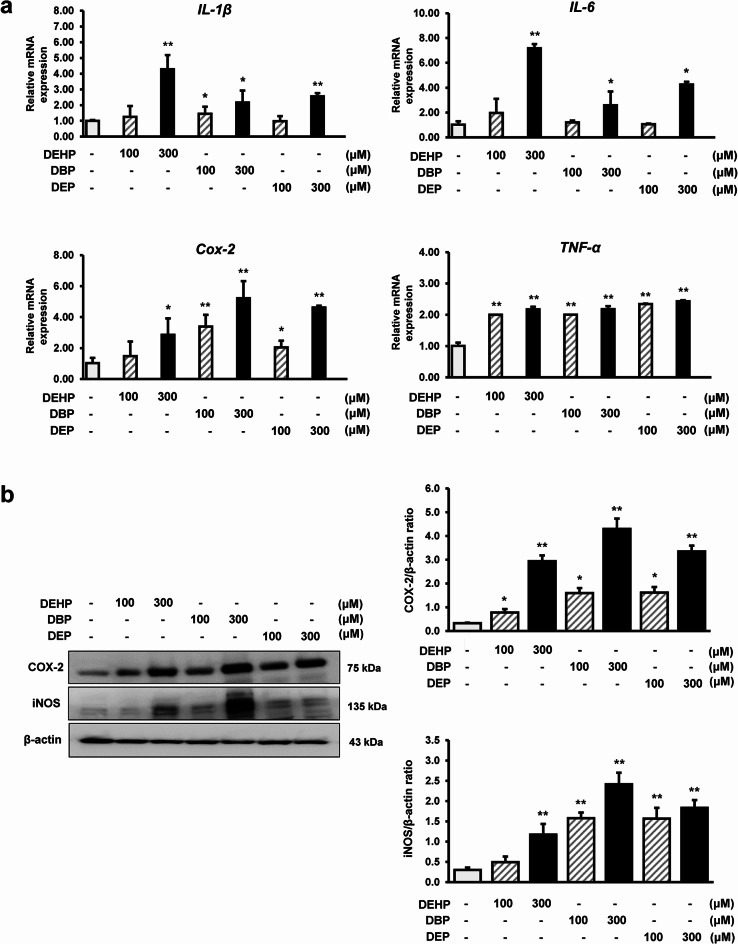
Inflammatory gene expression in RAW264.7 macrophages exposed to DEHP, DBP, and DEP. (**a**) mRNA expression of *IL-1β*, *IL-6*, *TNF-α*, and *COX-2* in RAW264.7 macrophages exposed to 0, 100, or 300 µM of phthalate for 24–48 h, using qRT–PCR. Data are presented as the mean ± standard deviation. *p < 0.05 and **p < 0.01 vs. vehicle control. Data were obtained from three independent experiments. (**b**) Protein levels of COX-2 and iNOS in RAW264.7 macrophages following treatment with 0, 100, or 300 µM of phthalates for 24–48 h, using western blot analysis. The expression levels of the proteins were quantified using ImageJ. β-actin was used as the internal control. Data are presented as the mean ± standard deviation. *p < 0.05 and **p < 0.01 vs. vehicle control. Data were obtained from three independent experiments

Phthalate exposure has been reported to induce oxidative stress and inflammatory responses. Here, treatment with 100 and 300 µM of DEHP, DBP, and DEP resulted in considerably increased *IL-1β*,* COX-2*, and *TNF-α* mRNA expression compared with that in the control group. *IL-6* mRNA expression was significantly upregulated upon treatment with 300 µM of DEHP, DBP, and DEP but not upon treatment with 100 µM DEHP, DBP, and DEP (Fig. 1A).

### DEHP, DBP, and DEP exposure on COX-2 and iNOS levels in RAW264.7 macrophages

COX-2 and iNOS are related to inflammation and produce mediators such as prostaglandins and NO. Treatment with 100 or 300 µM of DEHP, DBP, and DEP significantly increased COX-2 and iNOS protein levels in a dose-dependent manner compared with that in the control group. However, treatment with 100 µM of DEHP did not significantly increase iNOS protein levels compared with that in the control group (Fig. 1B).

### NAC ameliorates DEP-induced inflammation in RAW264.7 macrophages

**Fig. 2:**
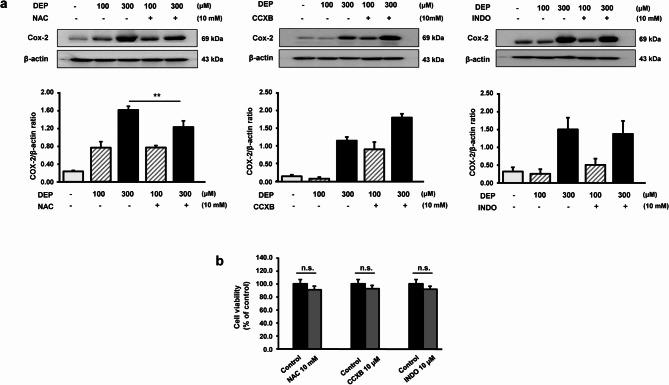
NAC ameliorates DEP-induced inflammation in RAW264.7 macrophages. (**a**) RAW264.7 macrophages were pretreated with CCXB (COX-2 inhibitor), INDO (COX inhibitor), or NAC (antioxidant) for 2 h, followed by DEP exposure. COX-2 protein levels were determined using western blot. The expression levels of the proteins were quantified using ImageJ. β-actin was used as the internal control. Data are presented as the mean ± standard deviation. *p < 0.05 and **p < 0.01 vs. vehicle control. Data were obtained from three independent experiments. (**b**) Viability of RAW264.7 macrophages exposed to CCXB, INDO, and NAC treatment. RAW264.7 macrophages were treated with 10 µM CCXB, 10 µM INDO, and 10 mM NAC for 48 h. NS, no significance

RAW264.7 macrophages were pretreated with CCXB, INDO, or NAC for 2 h, followed by DEHP, DBP, and DEP exposure. COX-2 levels were quantified using western blotting. As shown in Fig. 2A, NAC pretreatment significantly decreased inflammation induced by 300 µM DEP, whereas pretreatment with CCXB and INDO pretreatment did not produce a similar effect. In addition, pretreatment with CCXB, INDO, and NAC did not attenuate DEHP- and DBP-induced inflammation (data not shown). As shown in Fig. 2B, CCXB, INDO, and NAC treatments did not significantly affect the viability of RAW264.7 macrophages.

### NAC reduces oxidative stress and inflammation in RAW264.7 macrophages exposed to DEP

**Fig. 3:**
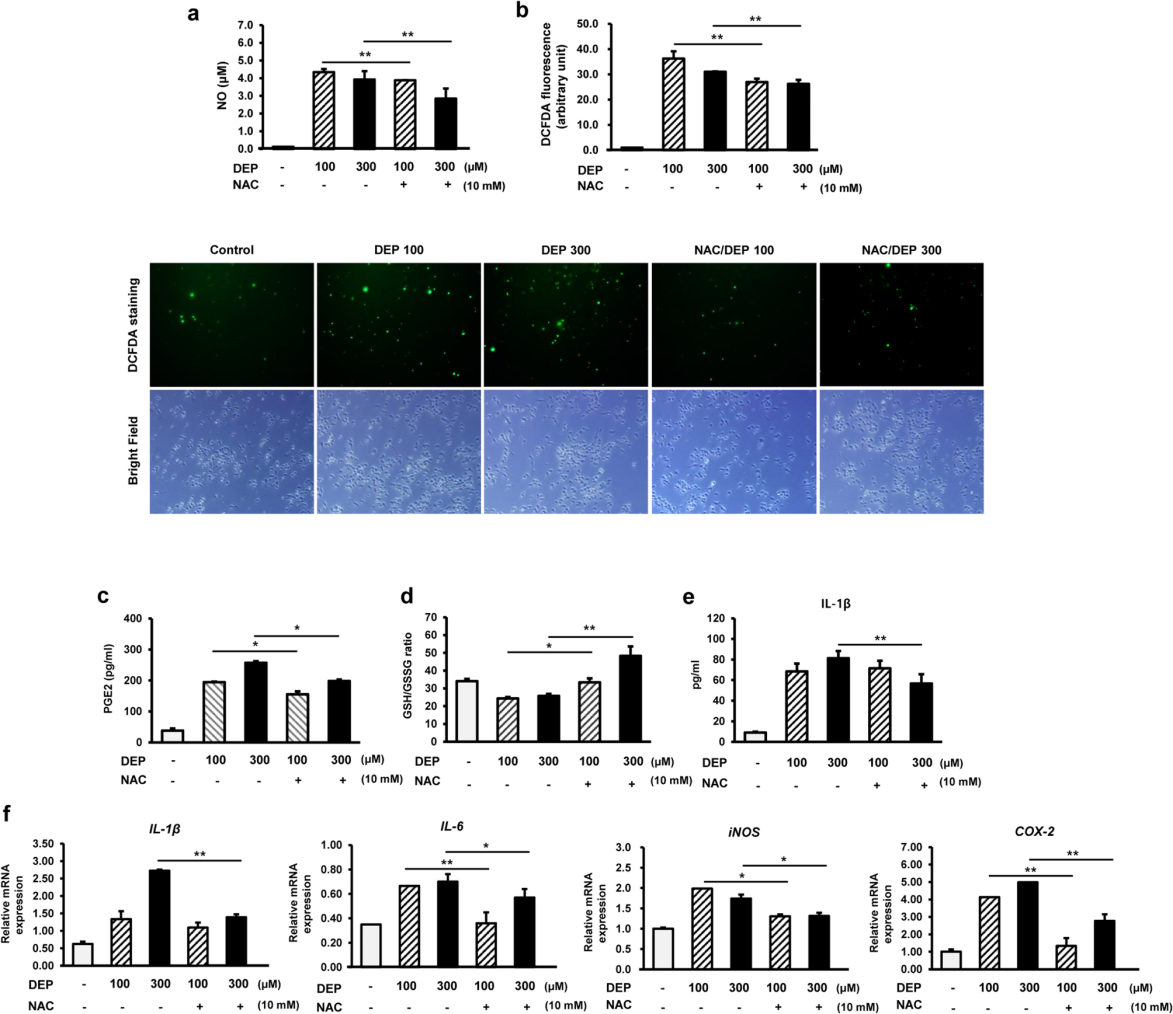
NAC reduces oxidative stress and inflammation in RAW264.7 macrophages exposed to DEP. NAC pre-treatment alleviated (**a**) NO, (**b**) ROS, (**c**) PGE2 level, (**d**) GSH/SSSG ratio, (**e**) IL-1β level, and (**f**) inflammatory-related gene expression, including *IL-1β*, *IL-6*, *TNF-α*, and *COX-2*, compared with those of DEP-induced RAW264.7 macrophages. Data are presented as the mean ± standard deviation. *p < 0.05 and **p < 0.01 vs. vehicle control. Data were obtained from three independent experiments

Inflammatory mediators, such as NO and PGE2, play important roles in the progression of inflammation. To investigate the inhibitory effect of NAC on DEP-induced inflammation in RAW264.7 macrophages, we examined the production of inflammatory mediators. NO, ROS, PGE2, and IL-1β levels were significantly lower in the NAC/DEP group compared with those in the DEP-treated group (Fig. 3A–C, E). NAC supports GSH synthesis and is a free radical scavenger. As shown in Fig. 3D, the GSH/GSSG ratio increased upon NAC treatment, alleviating the DEP-induced decline in GSH levels. Additionally, qPCR was performed to examine the effect of NAC pretreatment on the mRNA expression of pro-inflammatory genes involved in DEP-induced inflammation. *IL-1β*, *IL-6*, *COX-2*, and *iNOS* mRNA levels were significantly lower in the NAC/DEP group compared with those in the DEP-treated group (Fig. 3F). Overall, these results indicate that NAC ameliorates DEP-induced inflammation in RAW264.7 macrophages.

### NAC regulates DEP-induced inflammation via the STAT and MAPK signaling pathways

**Fig. 4:**
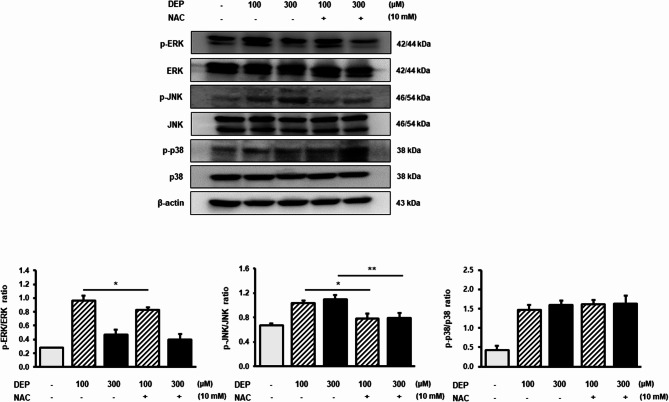
Effect of NAC on MAPK pathway proteins in DEP-induced RAW264.7 macrophages. Western blotting analysis was performed to determine the expression of phosphorylated ERK, JNK, and pp38 protein levels in RAW264.7 macrophages treated with NAC pretreatment following DEP-induced inflammation. The expression levels of the proteins were quantified using ImageJ. β-actin served as the loading control. Data are presented as the mean ± standard deviation. *p < 0.05 and **p < 0.01 vs. vehicle control. Data were obtained from three independent experiments

Next, we explored the molecular mechanisms underlying the NAC inhibition of DEP-induced inflammation in RAW264.7 macrophages using western blotting to analyze the expression of MAPK and STAT pathway-related proteins (p-ERK, p-JNK, p-p38, p-STAT1, and p-STAT3). The levels of p-ERK were significantly decreased in the NAC/DEP 100 µM group compared with those in the DEP-treated group, and p-JNK protein levels were significantly decreased in the NAC/DEP 300 µM group compared with those in the DEP-treated group (*p* < 0.05). However, the levels of p-p38 were decreased in the NAC/DEP group compared with those in the DEP treatment group, but the decrease was not statistically significant (Fig. 4).

**Fig. 5:**
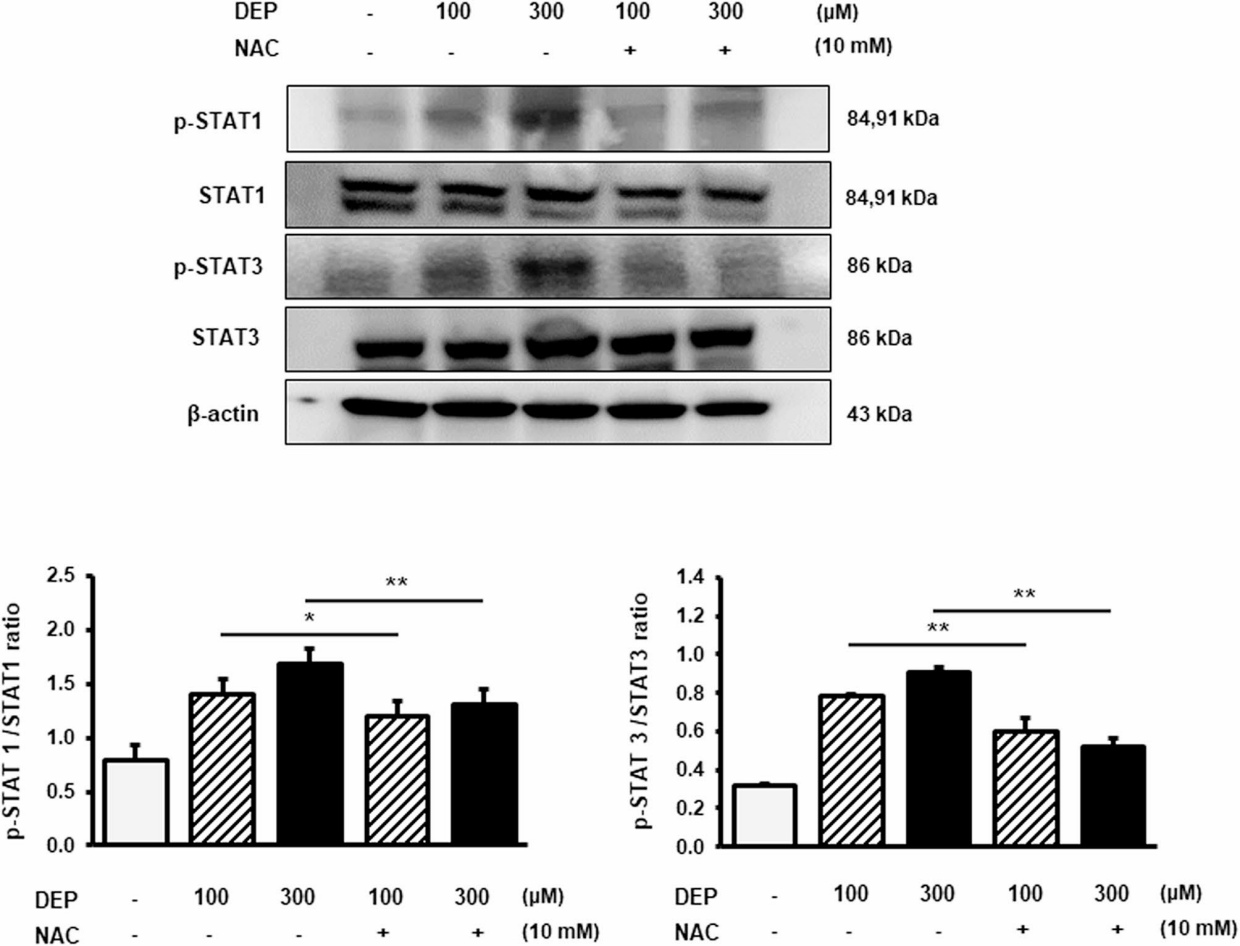
Effect of NAC on expression of the STAT1/3 pathway in DEP-induced RAW264.7 macrophages. Western blotting analysis was performed to determine the expression of phosphorylated STAT1 and STAT3 protein levels in RAW264.7 macrophages treated with NAC following DEP-induced inflammation. The expression levels of the proteins were quantified using ImageJ. β-actin served as the loading control. Data are presented as the mean ± standard deviation. *p < 0.05 and **p < 0.01 vs. vehicle control. Data were obtained from three independent experiments

As shown in Fig. 5, p-STAT1 and p-STAT3 protein levels were significantly decreased in the NAC/DEP (100 and 300 µM) group compared with those in the DEP-treated group (*p* < 0.05).

Therefore, NAC suppressed inflammation by blocking the phosphorylation of MAPK/JNK and STAT1/3 pathway proteins in RAW264.7 macrophages.

## Discussion

In this study, we established an inflammation model using DEP-stimulated RAW264.7 macrophages and elucidated the anti-inflammatory effect and underlying molecular mechanism of NAC. NAC pretreatment decreased pro-inflammatory cytokines and inflammatory mediators, including NO and PGE2, accompanied by decreased ROS production, in RAW264.7 macrophages exposed to DEP. Additionally, our results show that NAC downregulates COX-2 via MAPK/JNK and STAT1/3 signaling in DEP-induced inflammation in RAW264.7 macrophages. Furthermore, we found that pretreatment with NAC considerably attenuated DEP-induced inflammation and oxidative stress in macrophages.

Phthalates are among the most common environmental organic pollutants, some of which are endocrine disruptors that bioaccumulate in the body or are rapidly metabolized and excreted in the urine [9, 22]. Phthalate-induced toxicity has been reported in human and animal studies, raising concerns about the risks of environmental exposure to phthalates [8]. Oxidative stress and inflammation are interrelated processes caused by a variety of factors [23]. Oxidative stress occurs owing to an imbalance in ROS production and results in several pathological conditions, including inflammation [24]. Several studies have reported that phthalate metabolites can influence ROS production and oxidative stress and promote the production of inflammatory cytokines [25]. Moreover, previous research on phthalates has shown that MEHP exacerbates the inflammatory response and that DIBP causes cell death in macrophages [26].

We evaluated the inflammatory effects of three commonly used phthalates in RAW264.7 macrophages. Our results show that treatment with 100 and 300 µM of DEHP, DBP, or DEP significantly increased COX-2 and iNOS protein levels and upregulated the mRNA expression of the inflammatory factors *IL-1β*, *IL-6*, *COX-2*, and *TNF-α* in RAW264.7 macrophages. Furthermore, the NAC and COX inhibitors CCXB and INDO were investigated in DBP-, DEP-, and DEHP-stimulated RAW264.7 macrophages to investigate the anti-inflammatory effects. NAC decreased COX-2 protein levels in 300 µM DEP-treated macrophages, confirming its anti-inflammatory effects.

NAC is a known anti-inflammatory and antioxidant agent inhibiting NO production in various cells [27]. NAC acts as a free radical scavenger and glutathione precursor, protecting tissues against oxidative stress-induced cell death [28]. Additionally, NAC has beneficial clinical implications for heart, kidney, and liver diseases [29]. For instance, a previous study reported that NAC inhibited DEHP-induced increase in ROS production in ovarian antral follicles and improved testicular antioxidant status in rats [30].

However, the inhibitory effects of NAC on DEP-induced damage in macrophages remain unknown. In this study, NAC pretreatment significantly suppressed the increase in NO and ROS production and upregulated the decrease in GSH levels in DEP-induced RAW264.7 macrophages. NO production increases oxidative stress, which is associated with decreased total GSH levels [31]. In this study, DEP-induced inflammation appears to be caused by increased oxidative stress due to diminished GSH levels, and NAC suppressed this effect. Therefore, these results suggest that NAC regulates increased GSH levels and inhibits NO production in DEP-induced RAW264.7 macrophages.

Furthermore, NAC pretreatment downregulated the mRNA expression of the inflammatory genes *IL-1β*, *IL-6*, *COX-2*, and *iNOS* in DEP-induced RAW264.7 macrophages. *IL-1β*,* IL-6*,* COX-2*, and *iNOS* were overexpressed following DEP administration, resulting in an inflammatory response. Pretreatment with NAC reduced the inflammatory genes and suppressed the inflammatory response. These results show that NAC can be used to regulate DEP-induced inflammatory responses. In addition, since COX-2 and iNOS can produce PGE2 and NO, respectively, our results confirm the inhibitory effect of NAC on the upregulation of COX-2 and iNOS expression induced by DEP. Overall, our results indicate that NAC can inhibit DEP-induced damage, primarily through pathways related to oxidative stress and COX/PGE2. Mechanistically, we speculate that NAC alleviates DEP-induced damage by downregulating the levels of inflammatory mediators, including PGE2, and suppressing inflammation through COX-2 downregulation.

MAPK (including ERK1/2, JNK, and p38) signaling pathways play an important role as regulators of inflammation and mediators of inflammatory cytokine production in various diseases [32, 33]. The STAT family proteins are among the earliest proteins associated with inflammation [17], and activated STATs translocate to the nucleus and influence the transcription of genes such as COX-2 and iNOS [18]. ROS plays an important role in regulating the expression of inflammatory genes by modulating STAT phosphorylation via the MAPK pathway [18]. Previous studies have reported COX-2 as a molecular target of JNK, and JNK-induced COX-2 is involved in neurological dysfunction [34]. In mouse alveolar macrophages, ERK was activated in response to LPS-stimulated inflammation, with kinetics similar to those of p38 activation [35]. Moreover, STAT1 and STAT3 are activated upon the release of IL-6 from stimulated cells [36]. Based on these data, we investigated whether NAC ameliorates DEP-induced inflammation through the MAPK and STAT pathways. We observed that NAC could effectively inhibit the phosphorylation of JNK and STAT 1/3 in 300 µM DEP-induced RAW264.7 macrophages. NAC also downregulated the transcription of IL-1β, IL-6, COX-2, and iNOS, as well as the corresponding DEP-induced expression. These results suggest that NAC inhibits transcription and translation of relevant inflammatory genes. Furthermore, NAC may exert its anti-inflammatory effects by interacting with oxidative stress and the MAPK/JNK and STAT1/3 signaling pathways to modulate inflammatory factors and mediators. These results were based on in vitro models, which limit their application in animals and humans. Therefore, animal and human studies are necessary to confirm the anti-inflammatory effects of NAC following exposure to phthalates.

## Conclusions

Our findings expand our understanding of the effects of NAC in RAW264.7 macrophages, revealing its potential use for preventing DEP-induced inflammation. Our results demonstrate that NAC inhibits inflammation-related genes and oxidative stress through activation of the MAPK/ JNK and STAT1/3 signaling pathways in RAW264.7 macrophages. To the best of our knowledge, this is the first study to report that NAC protects against the effects of DEP in macrophages. Although our observations require in vivo studies, NAC may be a promising candidate for preventing the side effects of DEP exposure.

## Electronic supplementary material

Below is the link to the electronic supplementary material.

Supplementary Material 1

Supplementary Material 2

Supplementary Material 3

Supplementary Material 4

## Data Availability

The dataset supporting the conclusions of this article is included within the article and its additional files.

## Abbreviations

ATCC: American Type Culture Collection
CCXB: Celecoxib
COX-2: Cyclooxygenase-2
DBP: Di-n-butyl phthalate
DCFDA: 2’,7’-dichlorofluorescin diacetate
DEHP: Di(2-ethylhexyl) phthalate
DEP: Diethyl phthalate
DMEM: Dulbecco’s Modified Eagle’s Medium
DMSO: Dimethyl sulfoxide
ELISA: Enzyme-linked immunosorbent assay
ERK: Extracellular signal-regulated kinase
FBS: Fetal bovine serum
GAPDH: Glyceraldehyde 3-phosphate dehydrogenase
GSH: Glutathione
GSSG: Oxidized glutathione
IL-1β: Interleukin-1 beta
IL-6: Interleukin-6
INDO: Indomethacin
iNOS: Inducible nitric oxide synthase
JNK: c-Jun N-terminal kinase
MAPK: Mitogen-activated protein kinase
NAC: N-acetylcysteine
NED: Naphthalene ethylenediamine
NF-κB: Nuclear factor kappa B
NO: Nitric oxide
PCR: Polymerase chain reaction
PGE2: Prostaglandin E2
PVC: Polyvinyl chloride
qRT-PCR: Quantitative reverse transcription polymerase chain reaction
RIPA: Radioimmunoprecipitation assay
ROS: Reactive oxygen species
SDS-PAGE: Sodium dodecyl sulfate-polyacrylamide gel electrophoresis
STAT: Signal transducer and activator of transcription
TNF-α: Tumor necrosis factor alpha
