# Supplementary figures and images for: N-actylcysteine inhibits diethyl phthalate-induced inflammation via JNK and STAT pathway in RAW264.7 macrophages
Source: BMC Mol Cell Biol. 2025 Apr 16;26:12. doi: 10.1186/s12860-025-00537-9 (PMC12001441; doi:10.1186/s12860-025-00537-9)

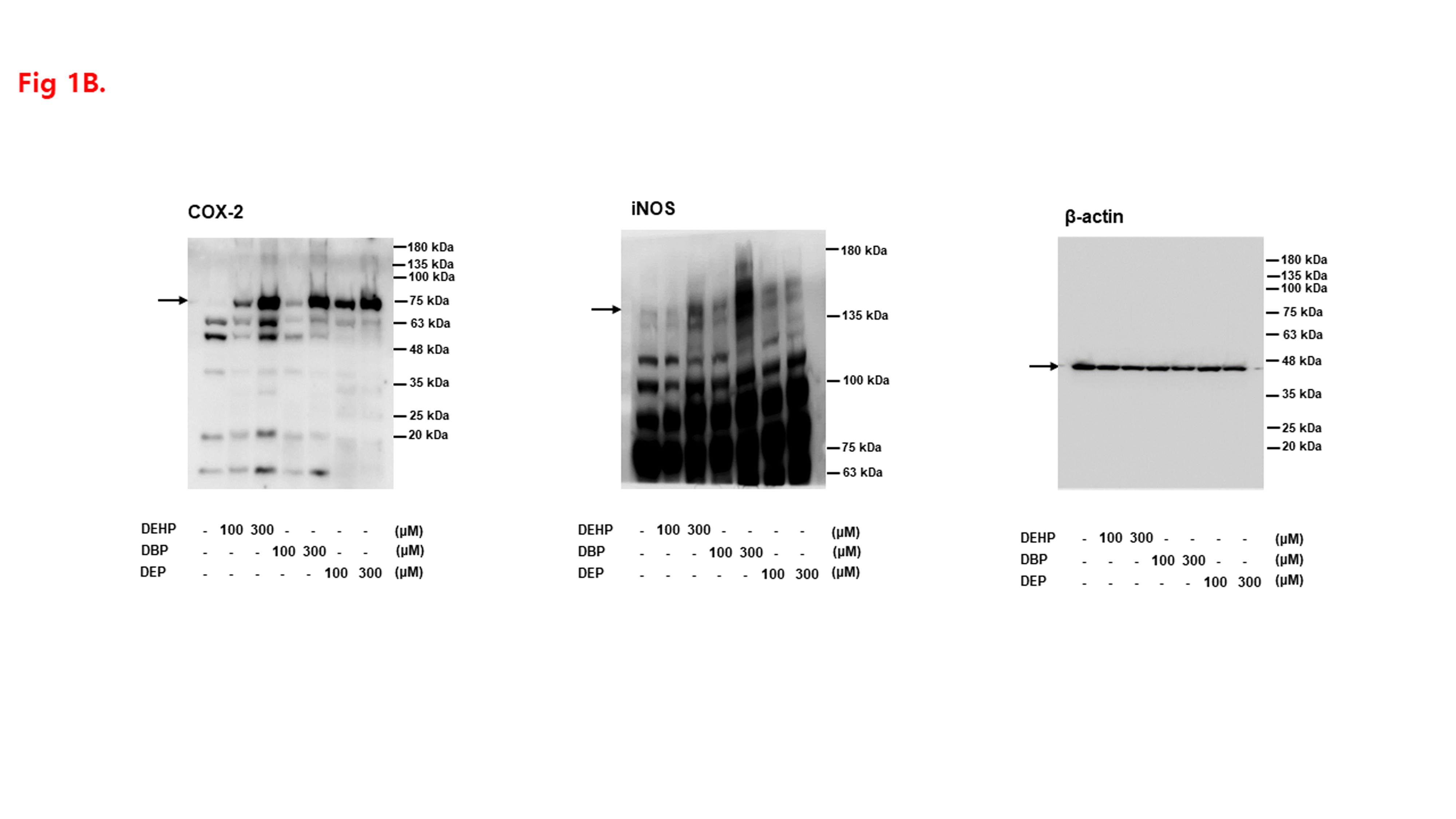

Supplement: Supplementary file 1 — Supplementary Material 1 [file 12860_2025_537_MOESM1_ESM.tif]

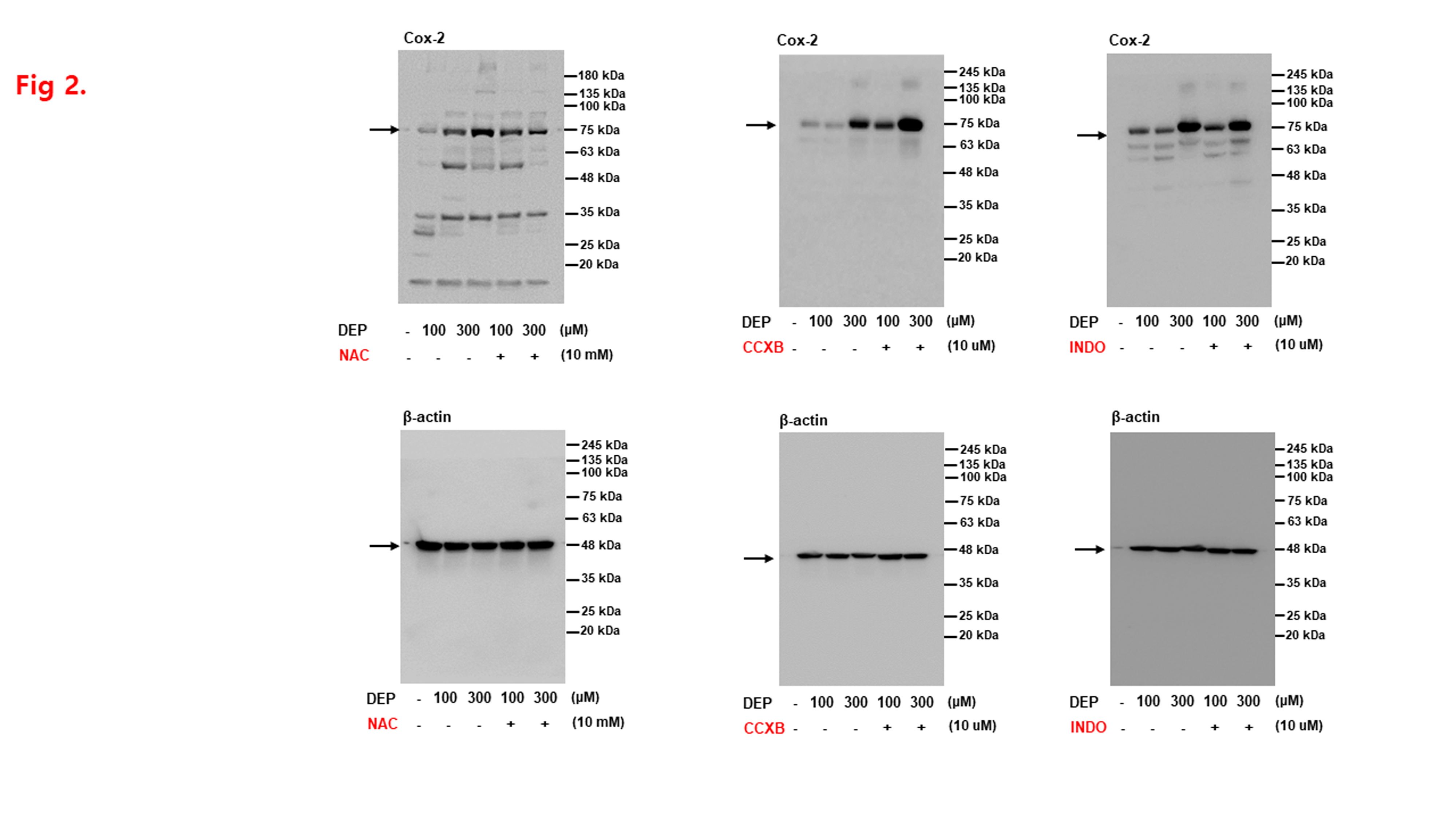

Supplement: Supplementary file 2 — Supplementary Material 2 [file 12860_2025_537_MOESM2_ESM.tif]

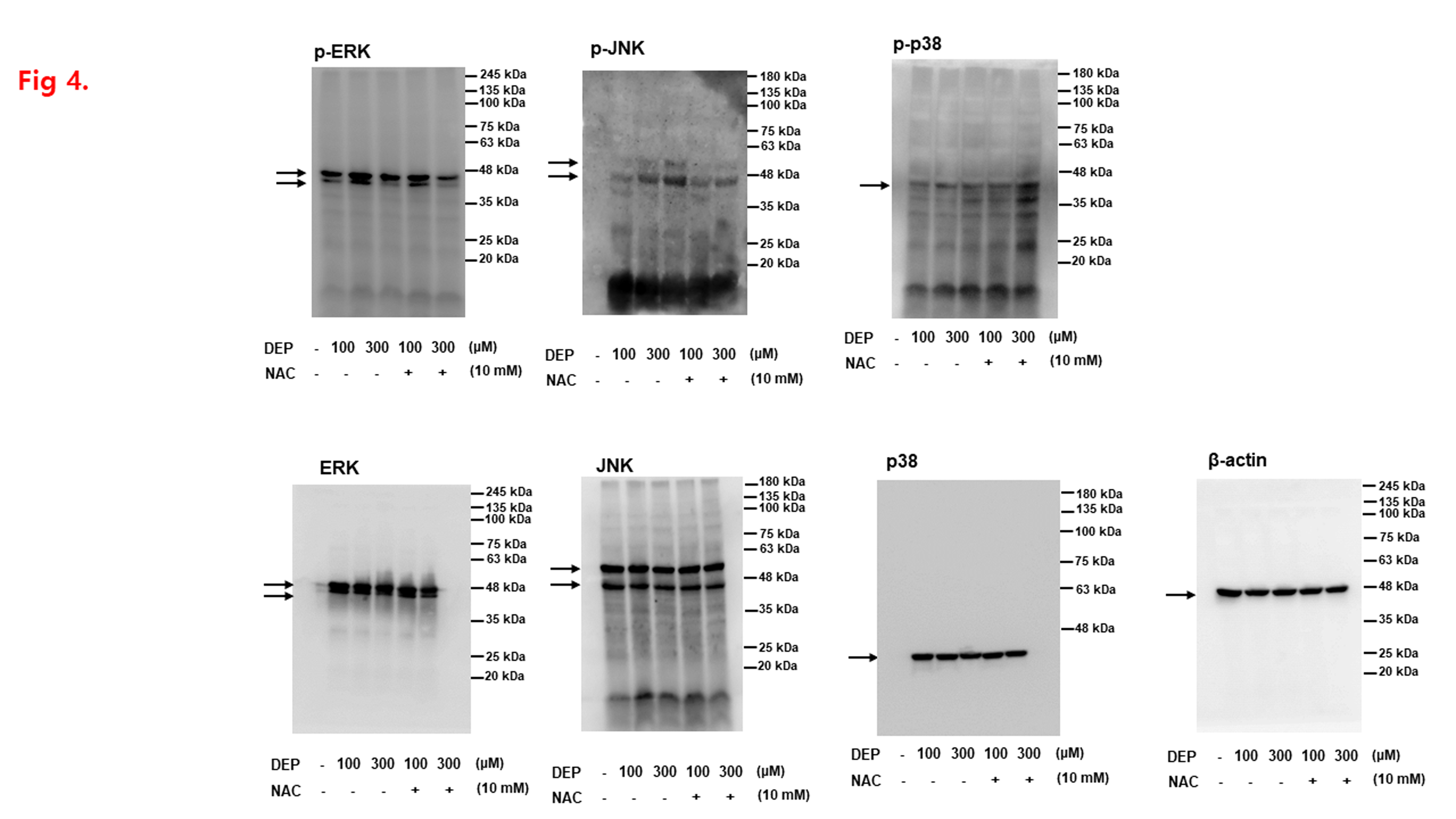

Supplement: Supplementary file 3 — Supplementary Material 3 [file 12860_2025_537_MOESM3_ESM.tif]

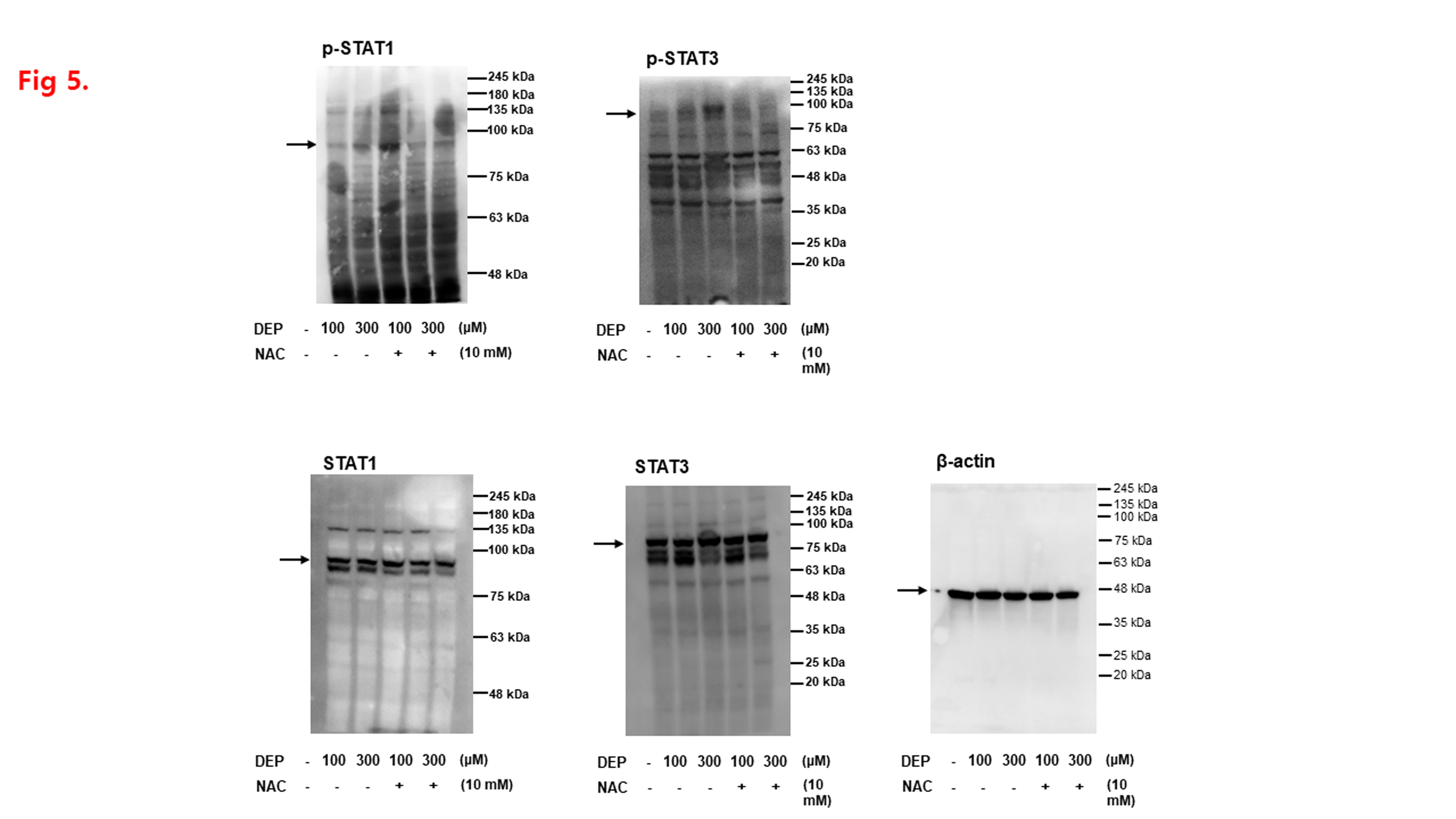

Supplement: Supplementary file 4 — Supplementary Material 4 [file 12860_2025_537_MOESM4_ESM.tif]
